# Understanding the Mechanisms of Change in the Supportive and Respectful Maternity Care Intervention in Sindh, Pakistan: Provider Perspectives

**DOI:** 10.9745/GHSP-D-23-00216

**Published:** 2023-12-22

**Authors:** Bilal Iqbal Avan, Waqas Hameed, Bushra Khan, Muhammad Asim, Sarah Saleem, Sameen Siddiqi

**Affiliations:** a Department of Population Health, London School of Hygiene & Tropical Medicine, London, United Kingdom.; b Department of Community Health Sciences, Aga Khan University, Karachi, Pakistan.; c Department of Psychology, University of Karachi, Karachi, Pakistan.

## Abstract

**Introduction:**

We conducted this qualitative investigation to explore the mechanisms of change in providing respectful care resulting from the supportive and respectful maternity care intervention (S-RMC) in Sindh, Pakistan.

**Methods:**

We applied the principles of realist evaluation methodology with a descriptive explanatory research design. We conducted in-depth interviews with 36 maternity care providers at secondary-level public health facilities where S-RMC was implemented for 6 months. The S-RMC broad components included capacity-building of maternity teams and systemic changes for improvements in governance and accountability within public health facilities. Data were analyzed using a deductive content analysis approach.

**Results:**

We identified mechanisms of change, categorized by the S-RMC components: (1) S-RMC training: insight into women's feelings and rights, realization of the value that nonclinical staff can play, understanding of team coordination, orientation in psychosocial components of maternity care; (2) assessment of women's psychosocial vulnerabilities: identification of women's differential needs beyond routine care to provide woman-centered care; (3) psychosocial support: effective engagement with women and within maternity teams and the customization of woman- and companion-focused care; (4) care coordination: improved coordination among clinical and nonclinical staff to provide personalized care and psychosocial support and proper handover to ensure continuity of care; (5) assessment of quality of care: identification of service gaps from women's feedback; and (6) performance review and accountability: monthly performance review meetings to establish team member communication, systematic awareness of the maternity team's performance and challenges, and implementation of collective corrective actions.

**Conclusion:**

Our findings pointed to S-RMC working along multiple pathways—and concertedly with various health system components—to enable positive processes and behavioral change in maternity teams.

## Abstract (precis)

The authors identify ways that a supportive and respectful maternity care intervention was implemented along multiple pathways–and concertedly with various health system components–to enable positive processes and behavioral change in maternity teams.

## Teaser

**Key Findings:**

- We identified several underlying mechanisms that were responsible for changes in providing supportive and respectful maternity care (S-RMC):
  - Strengthening providers' capacity of RMC by replacing existing harmful practices with behaviors designed to provide respectful care and psychosocial support helped providers better understand women's needs and preferences and better engage with them.
  - Revitalizing the notion of the maternity team to include all clinical and nonclinical staff as having a role in providing personalized care and support improved the continuity of care and staff communication.
  - Soliciting women's feedback about their birthing experience and embedding S-RMC in the existing performance review mechanism helped providers understand gaps and enabled them to support each other in implementing corrective actions.

**Key Implications:**

- Beyond assessing the effectiveness of an intervention that promotes respectful maternity care and a positive birthing experience, understanding the mechanisms of change is vital to inform the scale-up strategy for embedding S-RMC components within the health system.
- Such evidence should be complemented by large-scale research on S-RMC effectiveness and competencies to enable its responsiveness to needs in diverse settings.

## INTRODUCTION

Disrespect, discrimination, abuse, and lack of emotional support during intrapartum care are prevalent in health systems in many low- and middle-income countries (LMICs).^1^ Such woeful experiences of obstetrical care deny women's right to be treated with dignity, integrity, and respect and are likely to have adverse effects on both women and newborns.^2^ Furthermore, studies suggest that women with sociodemographic (e.g., primigravida or poor) and health-related (e.g., disabilities or mental illness) vulnerabilities face a higher risk of mistreatment.^1^^,^^3^^–^^5^ Mistreatment during childbirth is considered a strong deterrent to facility-based childbirth.^2^

In response to this global issue, the World Health Organization (WHO) published a revised framework for improved maternal and newborn care that placed special emphasis on women's experiences of care (in terms of dignity, effective communication, and emotional support).^6^ More recently, WHO released a set of evidence-based policy recommendations that defined respectful maternity care (RMC) as a combined construct of supportive and dignified care, emphasizing that all women are entitled to dignity, privacy, and confidentiality; be free from harm, mistreatment, and coercion; and receive continuous (psychosocial) support during labor and childbirth.^7^

The release of WHO's framework and guidelines galvanized action and research on RMC to ensure that all women are treated with respect and dignity during maternity care. Particularly in LMICs, several multidimensional interventions have been tested, including RMC training,^8^ community workshops, maternity open days, and quality improvement processes.^9^ These interventions showed a reduction in the incidence of mistreatment during childbirth in facility-based settings.^9^ Yet, these interventions faced several challenges: unsustainability of the demonstrated effect because of concerns around inadequate integration within the health system, lack of acceptability and feasibility in low-resource settings, lack of focus on inclusive care for dealing with women with preexisting psychosocial vulnerabilities, and a predominate focus on reducing disrespect and abuse^9^ while neglecting the component of supportive care.

Considering the need for an innovative intervention that is embedded within the health care system as a routine component of quality maternity care,^9^^–^^11^ we developed a theory-driven service delivery package to promote a culture of supportive and respectful maternity care (S-RMC) in public health systems using the principles of human-centered design.^12^ The intervention was implemented at public health facilities in southern Sindh, Pakistan, and the broader effect of the intervention on women's experiences of mistreatment has been reported elsewhere.^13^

This article draws lessons from implementing the S-RMC intervention at 6 public health facilities. Generally, information is limited on service providers' experiences of and reactions to S-RMC interventions and related factors affecting implementation.^10^ We believe this study will enhance understanding of the challenges and opportunities that surround the implementation of S-RMC interventions within health care systems and that its evidence base will contribute to the design and implementation of future S-RMC initiatives in low-resource settings.

We believe this study will enhance understanding of the challenges and opportunities of implementing S-RMC interventions within health care systems.

## THE S-RMC INTERVENTION

Our intervention involved a participatory, consensus-driven process of working closely with maternity teams, theoretically underpinned by the COM-B framework (capability, opportunity, and motivation drivers for respectful maternity behavior).^12^^,^^14^^,^^15^ The intervention components included capacity-building of maternity teams and systemic changes for improvements in governance and accountability within public health facilities (Table 1). The integration of psychosocial support in routine maternity care to address the needs of birthing women and their companions was a unique feature. The technical content of the S-RMC training handbook drew from WHO's intrapartum care guidelines^7^ and Mental Health Gap Action Programme materials for maternity settings^16^ and contextual evidence.^17^ Details of the intervention design, implementation, and effectiveness have been previously published.^12^^,^^13^ A member of the existing clinical maternity team—identified as a focal person called the “mental health first aider”—was made responsible for overseeing the implementation of S-RMC components in routine intrapartum care.

**TABLE 1.** Components and Subcomponents of the S-RMC Intervention in Sindh, Pakistan

Component	Description
**1. Building capacity**	
S-RMC training	Training (3 days) for all staff (clinical, nonclinical, and administrators) on: Leadership and team-based maternity care Supportive and dignified maternity care Clarification of professional values to practice S-RMC Ethical, rights-based, and woman-centered care Psychosocial support and its implementation Operationalization of S-RMC strategy^a^
**2. Improving governance and accountability mechanisms**	
Front-end operationalization of S-RMC: Activities and/or processes that directly engage pregnant women and/or their companions	
Assessment of women's demographic and psychosocial vulnerabilities	S-RMC activities and processes embedded within health facilities Orientation of women and companions about their rights and responsibilities and available support Register introduced for screening for psychosocial vulnerabilities (e.g., anxiety, depression, disability) and other sociodemographic vulnerabilities (e.g., poverty, lack of education or companionship, minority religion/caste)
Benchmarking respectful care and psychosocial support	Respectful care: avoiding violence, inclusive care, sharing information for informed decision-making, confidentiality, good rapport with pregnant woman, use of job aids Supportive care (i.e., psychosocial support): environmental support (cleanliness, privacy) and individual support (psychoeducation regarding needs and stressors, reduced stress, strengthened social support via companion engagement, and promoted daily scheduling and lifestyle changes)
Care coordination	For pregnant women: sharing of women's personalized preferences and needs with each team member For staff: addressing of psychosocial needs of staff
Assessment of quality of care	Periodic assessment of women's experiences of S-RMC through exit interviews and complaint management system
Back-end operationalization of S-RMC: Managerial, administrative, and information-system activities and/or processes linked to the maternity team	
Enhanced management information system	Consolidation of S-RMC-related data (e.g., vulnerability assessment and women's feedback and complaints)
Performance review and accountability	Introduction of periodic (monthly) performance review meetings, including discussion on S-RMC performance and taking actions Identification of S-RMC carer-of-the-month
Continuous professional development	On-the-job training and refreshers to staff by mental health first aider, a clinical member of the maternity team at each health facility

Abbreviation: S-RMC, supportive and respectful maternity care.

a The research team devised a customized strategy to operationalize S-RMC in routine intrapartum care.

In this post-intervention assessment, we sought to explore maternity staff and providers' perspectives on the mechanisms of change in providing respectful care resulting from the implementation of the S-RMC intervention and gather their recommendations for embedding S-RMC within health care facilities.

## METHODS

### Study Design and Setting

We applied the principles of realist evaluation methodology with a descriptive explanatory research design.^18^ This is a theory-driven evaluation method, which is increasingly used to study the implementation of complex interventions within health systems, including in LMICs.^18^ It is driven by the questions: what works, how, for whom, in what circumstances, and to what extent?^19^ In this study, we identified the mechanisms for each of the subcomponents of the S-RMC intervention package, except for “continuous professional development” due to a shorter implementation period. Furthermore, perspectives regarding “enhanced management information system” were covered in the component of “assessment of women's demographic and psychosocial vulnerabilities.” Data were collected through in-depth individual interviews. The study sites were secondary-level public health facilities that provided basic emergency obstetric and newborn care services and where S-RMC had been fully operative for 6 months.^12^^,^^17^ These health facilities were located in adjacent districts—Thatta and Sujawal—in Sindh, Pakistan. Thatta and Sujawal have a combined population of 1.7 million—almost 90% of whom live in rural areas^20^—and rank low on human development index strata, with only 17% women who are literate.^21^

### Study Participants and Sampling

We interviewed clinical staff, nonclinical support staff, and administrators who had undergone S-RMC training and worked in the maternity section during the 6-month intervention phase. Clinical staff included obstetricians/gynecologists, general physicians, nurses, midwives, and lady health visitors. Nonclinical staff included traditional birth attendants/aayas (attendants), sweepers, security guards, and ward boys (in district headquarters hospitals). Administrators included medical superintendents. We included nonclinical staff because their involvement in service provision and interaction with pregnant women is inevitable in our context, just as in other LMICs. Nonclinical staff play an important role due to their routine interaction with women and their birth companions.^17^ We selected study participants from a list of 120 maternity staff who attended a 3-day S-RMC training course. After consulting with health facility administrators, we omitted the names of maternity team members who were no longer part of the maternity section at the time of data collection. Forty participants were purposively selected by designation, according to a predetermined quota from each health facility (Table 2).

**TABLE 2.** Characteristics of Participants in the Qualitative Study of the Supportive and Respectful Maternity Care Intervention in Sindh, Pakistan

Characteristics	No. (%) (N=36)
District	
Thatta	20 (56)
Sujawal	16 (44)
Sex	
Female	29 (81)
Male	7 (19)
Age, mean (±SD), years	39 (±7.7)
Health worker cadre	
Clinical staff	
Obstetrician/gynecologist (doctor)	4 (11)
Nurse/midwife/lady health visitor	15 (42)
Nonclinical staff	
Traditional birth attendant/aaya	7 (20)
Cleaner and janitorial staff	3 (8)
Security guard/ward boy	2 (5)
Health managers	
Medical supervisor/medical superintendent	5 (14)
Total experience, mean (±SD), years	10.2 (±8.4)
Working shift	
Morning	21 (58)
Evening	5 (14)
Night	6 (17)
Others (rotation, full day, special days)	4 (11)

Abbreviation: SD, standard deviation.

### Interview Guide

We developed a semistructured interview guide to explore staff perspectives regarding the S-RMC intervention package and to understand the challenges service providers faced while implementing its different components and subcomponents (Supplement). Interview guide development used the realist evaluation approach.^18^ To understand how context influences intervention processes or mechanisms, we compiled a set of questions for each S-RMC component. We also included additional probing questions based on field observations and feedback received from maternity teams during the intervention period.

### Data Collection and Management

Data were collected from September to November 2022. Thirty-six semistructured in-depth interviews were successfully completed (despite repeated attempts, 4 potential participants could not be interviewed due to their busy schedules). About half of the interviews were conducted by research investigators (WH, BK, and MA) and half by a trained sociologist who had prior qualitative research experience related to maternal and reproductive health and had accompanied investigators during the initial interviews. Each interview was conducted in person in the local languages (Sindhi and/or Urdu) and in a separate room at the health facility with only the researcher(s) and interviewee present. All study participants gave their informed written consent. Interviews were audio-recorded with the study participants' permission. After each interview, the researcher was debriefed by trained data collectors, and the participant's responses were discussed. The interviews lasted 60−75 minutes, on average. Data collection concluded when the research team found that no new information was being elicited from the study participants.

### Data Analysis

All recorded interviews were transcribed by trilingual linguists (Sindhi/Urdu/English) with experience in translating health-related interview transcriptions. A deductive content-analysis approach was used to analyze the data.^22^ This top-down approach analyzes the data with several preconceived themes based on theory or an existing framework.^23^ We used the S-RMC implementation framework for data analysis (Table 1). The data analysis process included a comprehensive review of all transcripts, training feedback from participants, and field notes according to preexisting S-RMC themes. The data were analyzed by 2 co-authors (WH, BK) through NVivo version 11.0. To ensure data reliability, the co-authors examined all transcripts a second time for verification by using feedback during S-RMC training, field notes, and audio recordings. At the first stage of analysis, the 2 co-authors (WH, BK) identified the relevant participant's quotes according to S-RMC intervention components through a detailed review of the transcripts. This preliminary analysis was compared with the themes and subthemes of other co-authors (MA, SS). Discrepancies and overlapping data were discussed, resolved, and approved by all authors. Discrepancies during this process were resolved with the mutual consent of co-authors through triangulating and validating the narratives of clinical and nonclinical participants about providing the components of S-RMC to women during pregnancy and childbirth. Similar and overlapping codes were omitted during preparation of the article.

### Ethical Approval

The study protocol, informed consent forms, and other appropriate documents were approved by the Ethics Review Committee of the Aga Khan University (Ref. ID: 2019-1683-5607) and Research Ethics Committee of the London School of Hygiene & Tropical Medicine (Ref. ID: 17886).

## RESULTS

We included 36 in-depth interviews in the analysis. Table 2 shows the sociodemographic characteristics of the participants. The mean age was 39 (±7.7) years, and participants had working experience of 10 (±8.4) years. Eighty percent were female, 53% were clinical staff, and about 58% worked the morning shift.

We describe the study participants' perspectives regarding different components of the S-RMC intervention in the following section. Table 3 summarizes the mechanisms of change identified in the intervention.

**TABLE 3.** Summary of Mechanisms of Change Resulting in More Dignified, Supportive, and Respectful Maternity Care in Secondary Public Health Facilities in Sindh, Pakistan

Component	Mechanisms of Change
S-RMC training	Insight into women's feelings and rights Realization of the worthiness of the role nonclinical staff can play Understanding how to operationalize team coordination Orientation in psychosocial component and its place within maternity care
Assessment of women's demographic and psychosocial vulnerabilities	Identification of women's differential (psychosocial) needs beyond routine care Provision of woman-centered care
Benchmarking dignified care and psychosocial support	Effective engagement with pregnant women and within maternity teams Customization of woman- and companion-focused care
Care coordination	Collective commitment of team to achieve personalized care Psychosocial needs also supported by nonclinical staff Continuity of service—proper handover
Assessment of quality of care	Identification of service gaps Empowerment of women within the health system
Performance review and accountability	Creation of a means of communication among team members Systematic awareness of the maternity team's performance and challenges Implementation of collective corrective actions

Abbreviation: S-RMC, supportive and respectful maternity care.

### S-RMC Training

All maternity team members acknowledged that the 3-day S-RMC training was very relevant to their routine work. The training helped staff realize the importance of women's feelings, personalized (nonclinical) needs, and expectations during childbirth, as well as how service providers can understand and address those needs in a systematic and nondiscriminatory manner. Nonclinical staff highlighted their sense of worthiness in their role of consoling pregnant women and their companions and thus positively influencing the childbirth process.

The training helped staff realize the importance of women's feelings, needs, and expectations and how they can understand and address those in a systematic and nondiscriminatory way.

*I felt a lot of change . . . now I feel as if I am helping someone . . . my practice has improved a lot after the training …* —Aaya, Thatta

A majority of staff members also highlighted an improvement in team coordination, interaction, and support for each other. Specifically, the janitorial staff reported a positive change in the attitudes and behaviors of clinical staff toward them. Staff across all cadres said that they not only felt a positive change in themselves but also observed such a change in the maternity team while providing care. Some clinical staff said that the training had motivated them emotionally to pay attention to women's personal needs, which they used to overlook in favor of clinical needs.

*The good thing is that we focus on the new aspects now which we didn't used to focus on much before … like our behavior with the patients, our attitude and understanding her problems, guiding them accordingly in a good way …* —Gynecologist, Sujawal

In terms of training content, most staff liked the psychosocial component. Considering nonclinical staff members' routine interaction with women and their companions, their inclusion in the training was also appreciated. Maternity teams offered several suggestions, such as including administrative staff, having periodic refreshers, and addressing language barriers.

*We learned how to screen which patient is vulnerable and needs more attention … how we should behave with the patient during labor so she feels comforted.* —Nurse, Sujawal

However, a few nonclinical staff members said that they could not express themselves well in the training because of the presence of senior clinical staff.

### Assessment of Women's Demographic and Psychosocial Vulnerabilities

The on-duty senior staff nurse who carried out the initial examination was responsible for screening women for vulnerabilities. Maternity staff found that the standard registers provided a systematic way of identifying women's psychosocial needs, which helped them to plan and provide care accordingly. They reported that women had never been asked such questions. Some staff members added that when women were asked these questions, they felt that service providers were giving them attention. The screening questions served as a means for the service provider to be polite, gentle, and respectful with sensitive women and avoid behaving in a way that might damage their self-esteem.

*Before, they never asked patients any depression or anxiety questions … to be very honest … this was out of the question … patients used to come, we provided treatment, and they went. When this register was introduced, then they began to ask the patients detailed questions … patients used to feel good because they thought that somebody was focused on them …* —Gynecologist, Sujawal

Because senior staff conducted the screening while they were engaged in other activities, it was difficult for them to fill in the register when client volume was high. Having a separate register compounded this challenge. To make the screening process manageable, participants suggested that recordkeeping either be done by a dedicated person or be evenly distributed among staff members and that new questions be embedded in existing registers.

*It became a lot easier to look after the patient … everything was written in detail, and point by point, to identify the patient's problem … We get to know in reality whether the patient has depression …* —Nurse, Thatta

*It was a good and novel thing… we were given different registers to fill in… but I faced many problems in filling those registers… because I was alone, and I also have to manage everything here.* —Nurse, Thatta

### Benchmarking of Dignified Care and Psychosocial Support

Service providers reported that when they listened to women and their companions and treated them with dignity, women spoke more openly/comfortably with staff, were cooperative, and adhered to given instructions. Improved interaction enabled maternity staff to address women's concerns and resolve issues more effectively, which, in turn, increased women's satisfaction. Participants reported improvements across different aspects of care, including maintenance of confidentiality, provision of nondiscriminatory care, reduction in physical or verbal abuse, and cleanliness of health facilities.

*Staff don't talk to patients disrespectfully now … or do patient check-ups without informing them. . . . We explain things politely to them … If she does not have a companion, then we tell the aaya to be with the patient and take care of her needs.* —Nurse, Sujawal

*We realized that, yes, patients are sensitive and they do have mental problems … we used to focus just on her [clinical aspects of] pregnancy … now we focus more on the mental condition of the patient … is she not well due to poverty and social stress, or are there some other issues which she cannot express? Sometimes the patient has discharge [bleeding] and she cannot tell you about it.* —Nurse, Thatta

In terms of psychosocial support, effective engagement with a companion emerged as a promising approach that benefited both service providers (reducing their burden through task-sharing) and women (having a companion of choice for support). Nonclinical staff (aayas or sweepers) were sometimes engaged to support pregnant women by consoling them or helping them walk when their companion was absent. Although this strategy helped build trust between women and service providers, occasionally, companions became overly demanding and pressured staff unnecessarily for immediate care.

*We now keep the companion with the patient … we tell her [companion] that her duties are to make the patient drink juice or water, make her eat something, do breathing exercise … mobilize the patient. There are not enough staff members in the government setup, and that's why we involve companions. It keeps the companion busy. They are good in this way, and it reduces our burden.* —Gynecologist, Thatta

The maternity team's efforts to practice supportive and dignified maternity care effectively were hindered by the unavailability of medicines, high client volume, and the stubbornness and rudeness of some women or companions whose demands for services were persistently unreasonable despite explanations.

*If the patient is severely anemic or if her BP [blood pressure] is very high, we tell them [companion] to take her to the tertiary level hospital. Suppose they don't agree to take her there: they say, why you are referring her… can't you control her BP…? In that case, we have to face many difficulties… sometimes we feel helpless due to this resistance from them.* —Gynecologist, Sujawal

### Care Coordination

Participants reported that improved coordination among staff members helped in meeting women's personalized needs. Maternity teams were now also discussing psychosocial needs (the results of vulnerability assessments) during case discussions. If no companion was present, nonclinical staff were sometimes engaged to support or assist pregnant women with different tasks. At 1 health facility, the maternity team revived a proper handover mechanism between shifts, which had not been practiced for a long time.

*Before, doctors used to say, this is not my work … this is your work … why should I do it … but after the training, this tension has stopped, and all the doctors, nurses and lower staff work together.* —Nurse, Sujawal

*Before, our shift ended at 2 pm, and there were no rules of taking over … Now we wait for the next shift of staff so that I properly give the other staff handover … it was not happening previously. A staff member from the other shift called her colleague and told her to stay for 10 minutes as she was on her way … coordination was improved a lot.* —Gynecologist, Sujawal

### Assessment of Quality of Care

Service providers reported the benefits of gathering women's experiences of maternity care at the time of discharge. Gathering women's feedback helped providers identify service gaps and gave women a sense of worthiness and empowerment. Service providers reported their reluctance to enter complaints in the client complaint register and their tendency to discuss the complaints and resolve them immediately and verbally. They reported several reasons for doing this, such as their fear of severe consequences from supervisors if a complaint had been lodged against them and concern that documenting complaints would jeopardize staff relationships because most team members had a good working relationship with their colleagues. Nonetheless, they were willing to keep the complaint register to hold them accountable for poor practices or mistreatment of women. To make the complaint process more effective, service providers suggested keeping the register with a neutral person outside the maternity section, having a complaints box, or introducing a toll-free central complaint management system (helpline) that women could call directly.

Gathering women's feedback helped providers identify service gaps and gave women a sense of empowerment.

*Some staff were reluctant to fill in the complaint registers as it may put coworkers in serious trouble, which may jeopardize their relationships.* —Gynecologist, Sujawal

*It [patient complaint] is important because it keeps staff on their toes. But I think the register should lie with MS [medical superintendent] office, or you can instead put a complaint box so patient could drop a complaint in the box.* —Nurse, Thatta

*This register should be there … because the staff know that the register is there … if they do anything wrong, then it will be written in the register, then they can face the issue from the management, or they have to submit an explanation.* —Medical superintendent, Thatta

### Performance Review and Accountability

Monthly performance review meetings, which were new for nearly all the study's health facilities, were deemed very useful in addressing operational issues, including staff behavior. Participants reported that numerous practical measures were taken because of these meetings. The meetings established an essential means of communication among maternity team members and provided a forum to raise awareness of the section's performance and challenges. At the monthly meetings, staff discussed S-RMC intervention reports and various issues (e.g., supplies stock-out, staff roster, staff workload and absenteeism, infrastructure problems, and client complaints), as well as corrective actions taken.

*Here [at health facility] we had a problem of patients' privacy … we discussed it, and curtains were installed for privacy… then there were washroom issues … now it got fixed … many things got implemented which clinical staff told us all about during the monthly meetings …* —Gynecologist, Thatta

Because the meetings occurred in the daytime during working hours, participation was usually restricted to senior clinical and administrative staff; often, night staff were unable to attend. A few nonclinical staff members said that they were not informed about meetings. Nearly all staff members expressed a willingness to continue the meetings even after the intervention period. Participants suggested that all key staff members from all shifts attend.

*There is much change … we also discuss whatever issues are there … we have also developed a WhatsApp group, and we also share everything on that group. . . .* —Medical superintendent, Thatta

*We have discussions in the meetings… people tell their issues openly and they are also resolved… and staff also get the guideline for what to do or not to do …* —Medical superintendent, Thatta

## DISCUSSION

This qualitative study aimed to understand the mechanisms of change resulting from an S-RMC intervention—an approach that upholds human rights and the best standards of clinical care as guiding principles of maternity services to address women's dignity, differential needs, and psychosocial support. Reflecting on the results, we discuss the mechanisms of change identified in the intervention within the health system and broader LMIC context. We also consider the implications of embedding such a complex intervention, given the strengths and limitations of the research.

### Building Staff Capacity to Provide Inclusive, Supportive, and Dignified Care

RMC is sometimes oversimplified in training programs by only focusing on selected abusive behaviors. However, the lack of abusive behaviors alone does not render maternity services respectful. Replacing existing practices with behaviors designed to provide care and support and to confer dignity is essential for aligning maternity services with recommendations for a positive birthing experience^7^ so that they are deemed fit for purpose. Several educational and training programs have focused on raising awareness about RMC among health care workers in Africa and Asia.^8^ However, because these programs did not link with the health system process, including routine supportive supervision, their narrow focus on training limited their effectiveness.

The capacity-building component of S-RMC was broadly based and multidimensional. It focused on the core concepts of inclusive, supportive, and dignified intrapartum care, emphasized how they are interlinked with woman-centered, ethical, and rights-based care; provided an understanding of psychosocial support; incorporated the skills needed to provide support for pregnant women and their companions and for maternity staff; and included the skills required to operationalize care coordination in health facilities to achieve inclusive, supportive, and dignified maternity care.^24^

Maternity teams trained in S-RMC reported that the training gave them a clear sense of women's rights and emotions and of how to provide adequate services, including psychosocial support, during maternity care. Moreover, the training helped them understand that achieving dignified care for pregnant women requires a collective and collaborative effort rather than just individual actions.

### Enabling Providers to Assess Women's Personal Characteristics, Preferences, and Needs Beyond Routine Care

Low socioeconomic status and inequity are among the fundamental reasons for the poor health of populations.^5^ Failure to design health services with an inclusive health care perspective exacerbates this effect.^25^ Therefore, making inclusive health care integral to universal health care packages represents a necessary paradigm shift in primary health care systems.^26^ Yet, unfortunately, such efforts in LMICs to make care inclusive of marginalized groups of women during maternity care have not gained momentum observed elsewhere.^27^

The lack of an inclusive perspective in maternity health services leads to women suffering unfair and abusive practices based on racial, ethnic, socioeconomic, and physical and mental health differentials.^5^^,^ ^28^ A unique feature of the S-RMC intervention was the introduction of measures and tools at the point of admission to the maternity unit that enabled the maternity team to identify women's personal characteristics, preferences, and mental health needs, which were not typically included. Maternity staff confirmed that assessing women's needs beyond routine care gave them the confidence to deliver customized, woman-centered care to facilitate a positive birthing experience. However, health systems in LMICs are grossly underfunded and understaffed. As such, implementing this change proved challenging and, as staff pointed out, difficult to sustain during busy peak hours. For our short-term study, separate registers were introduced for recordkeeping. These screening questions could be added to the existing recordkeeping registers instead of using parallel recordkeeping to ensure that staff routinely conduct these assessments in the future.

### Equipping Staff With Psychosocial Support Skills to Provide Customized Care

Compassion is fundamental to medical care and affects pregnant women's maternity experiences.^29^ However, there is still no consensus on theoretical and attributional parameters and the competencies required to practice compassion. Moreover, the epidemiological means to operationalize and assess compassion are lacking.^30^ We realized this challenge very early in the development of our S-RMC intervention. Service providers perceived compassion as an emotion-based construct rather than a skills-based one. A primary manifestation of compassion is psychosocial support. Empathy and forming a caring relationship with the woman are the basis of compassion, which is the precursor to psychosocial support. Psychosocial support is a well-defined, operationalized phenomenon for health systems.^12^ A seminal example is WHO's Mental Health Gap Action Programme initiative, which seeks to address the lack of care for people suffering from mental health issues by technically empowering general health care providers who work in nonspecialized health care settings in LMICs. The effectiveness of its psychosocial elements is well established across diverse contexts.^31^^,^ ^32^ Ours was the first research initiative to systematically and rigorously adapt the Mental Health Gap Action Programme, especially its psychosocial components, for the maternity care setting in LMIC health systems.^31^ After acquiring psychosocial skills as part of the S-RMC project, maternity staff were able to identify fundamental mechanisms of change. They felt better equipped—with understanding and skills—to engage effectively with pregnant women and their maternity team colleagues. Focusing on a woman's mental, emotional, and social needs played a significant part in being able to customize maternity care to her specific needs. Furthermore, this led to attenuating disrespectful and abusive behaviors among service providers, resulting in better care provision.

### Making Personalized Care the Responsibility of Both Clinical and Nonclinical Staff

Coordinating the delivery of high-quality services entails deliberately organizing care provision tasks based on women's individual needs and all the maternity stakeholders regularly communicating.^33^ In a coordinated model of care, close collaboration among members of a multidisciplinary maternity team is a prerequisite for women's physical and psychological well-being and, consequently, their caring engagement with their babies. However, more evidence is needed about the effectiveness of the implementation strategies for this model, especially in overcoming professional and cultural hierarchies within the public maternity care system in LMICs.

A core process for initiating S-RMC was reviewing the concept of the maternity care “team.” We developed the concept of the team both horizontally and vertically. In the conventional view of the team, the clinical staff in the labor room are the core team responsible for client care and may consult clinical staff from other disciplines if a woman's condition requires it (vertical coordination). Providing psychosocial support for pregnant women by linking with nonclinical staff in the maternity ward (horizontal coordination) has been limited. By revisiting and expanding the notion of the team, all maternity ward team members felt a collective responsibility toward providing personalized care, which meant that psychosocial support took center stage in the process of care. Nonclinical staff were trained and expected to support women and their companions, especially where existing staff were already overburdened. An unexpected positive outcome of this was that the clinical team felt more responsible for the continuity of care of women, resulting in improved handover notes and communication about the woman's status during staff changeover at the end of shifts.^34^

### Soliciting Women's Feedback on Experiences of Care to Improve Quality

Maintaining and improving the quality of maternity services are ongoing processes, relying heavily on women's feedback about the positive birthing experience and the degree of their satisfaction with the services they received.^35^ Our approach was to solicit feedback at the moment women were discharged from the maternity unit. Feedback is generally valid and reliable at the end of maternity services when women feel reasonably confident that their honest feelings will have no negative repercussions for them. Service providers reported that this process empowered women within the health system and helped women feel that their feedback enabled maternity ward staff and hospital administrators to identify shortcomings in maternity care and was instrumental in improving maternity care services.

### Integrating Routine Performance Review Mechanisms to Develop Shared Staff Responsibility and Accountability

Health policymakers, administrators, and service providers increasingly accept that disrespectful and abusive maternity care is pernicious and endemic across health systems. Remedial efforts have focused on measurement, orientation, and training, yet the critical factors of accountability and support for behavior change are missing from the discourse around such interventions.^36^

S-RMC was embedded in the existing performance review mechanism in maternity wards and hospital systems. Service providers reported that this approach helped them develop a shared understanding of shortcomings in their practices and enabled them to support each other in implementing corrective actions.

Providers reported that embedding S-RMC in the existing performance review mechanism helped them develop a shared understanding of shortcomings in their practices and enabled them to support each other in implementing corrective actions.

### Contextualizing the S-RMC Intervention for Effective Implementation

Overall, the maternity team members shared a positive opinion about the different S-RMC intervention components and the changes they brought to routine care provision in enhancing their understanding of respectful care as well as their impact on the experiences of birthing women and their companions. We found complementary views expressed by different cadres of maternity teams regarding mechanisms of change, and no conflicting perspectives were documented.

Several underlying factors were identified that triggered changes in the provision of routine maternity care. Among these, staff considered S-RMC training, performance review, and accountability mechanisms the most influential drivers of change. The training not only enhanced knowledge but also fostered a realization of women's psychosocial needs, especially among team members with limited or no baseline knowledge. In the absence of existing practices, the performance review and accountability measures improved communication among team members and provided a platform to reflect on routine operations, enabling them to take corrective actions. All the change mechanisms in the S-RMC program offer a holistic approach to enhancing care provision at health facilities. The concept of team-based care drove the effect through a shared understanding of the S-RMC and collective efforts. However, for future efforts, it is pertinent to note that due to the variations in health care systems across countries, replication of this S-RMC intervention requires formative research for contextual adaptation to optimize relevance, operationalization, and effectiveness.

### Limitations

The principal caveat to our findings is that they represent the understanding of maternity teams and hospital administrators of the mechanisms of change based on the short-term implementation of S-RMC that was conducted immediately after the S-RMC training. Therefore, the findings do not indicate whether these behavioral changes would be sustained over the long term. Because the views gathered in the study were based on a short-term (6-month) implementation, the configuration of changes and mechanisms might be modified or differ altogether after long-term implementation. The sustainability drivers of health system interventions would also need to be considered in the long run. S-RMC is a behavior change intervention that is complementary to, but not a substitute for, a well-functioning health system. Adequate funding, staffing, and equipment will always be primary prerequisites for quality care services. Furthermore, the perspectives regarding mechanisms of change identified in our research are context specific. Mechanisms of change may be different in other contexts with different constellations of maternity service providers. Future research should also explore the effectiveness of interventions from the perspective of care recipients, including birthing women and their companions.

## CONCLUSION

In this study, we assessed the mechanisms that underlie and are responsible for a change in the provision of respectful care against the 6 core components of the S-RMC intervention. Based on this assessment, S-RMC works along multiple pathways and concertedly with various health system components to enable positive processes and behavioral change in maternity teams. Beyond its usefulness in assessing the effectiveness of an intervention that promotes RMC and a positive birthing experience, understanding the mechanisms of change is vital to inform the scale-up strategy for embedding S-RMC within the health system. Nevertheless, such evidence should be complemented by large-scale research on S-RMC effectiveness and competencies to enable it to be responsive to needs in diverse settings.

## Supplementary Material

GHSP-D-23-00216-supplement.pdf

## Notes

**Peer Reviewed**

**First published online:** November 20, 2023.
