# Supplementary material for: Understanding the Mechanisms of Change in the Supportive and Respectful Maternity Care Intervention in Sindh, Pakistan: Provider Perspectives
Source: Glob Health Sci Pract. 2023 Dec 22;11(6):e2300216. doi: 10.9745/GHSP-D-23-00216 (PMC10749650; doi:10.9745/GHSP-D-23-00216)
Supplement: GHSP-D-23-00216-supplement.pdf [file GHSP-D-23-00216-supplement.pdf]

Supplement to: Avan BI, Hameed W, Khan B, Asim M, Saleem S, Siddiqi S. Understanding the mechanisms of change for the supportive and respectful maternity care intervention in Sindh, Pakistan:provider perspectives. *Glob Health Sci Pract.* 2023;11(6):e2300216. https://doi.org/10.9745/GHSP-D-23-00216

In-depth interview guide for health facility staff

S.no	Question	Responses
**General information**		
101	Date of interview	|___|___| / |___|___| / |___|___|
102	Name of district	1 Thatta<br>2 Sujawal
103	Name of health facility	
104	Name of interviewer	
105	Cadre of healthcare provider	1 Clinical staff<br>2 Non-clinical staff<br>3 Health managers
106	Sex	1 Male<br>2 Sujawal
107	Age in years	|___|___| Years
108	What is your designation in this hospital?	1 Women medical officer<br>2 In-charge of Obs/Gyne<br>3 Nurse<br>4 Midwife<br>5 Cleaner<br>6 Technician<br>7 Aaya<br>8 Security guard<br>9 Medical superintendent<br>10 Information management officer<br>99 Others specify ____________________
109	How long have you been working in this health facility	______ months _______ years
110	What is your educational qualification?	
111	What is your total professional experience?	______ years
112	Do you work in the morning shift or evening?	1 Morning<br>2 Evening<br>3 Night<br>4 Specific days<br>5 Rotation
113	Can you tell me your official timings of the work?	**Day time:**<br>From |___|___|:|___|___| To |___|___|:|___|___| **Evening time:**<br>From |___|___|:|___|___| To |___|___|:|___|___|

114	Did you read out the informed consent form to the respondent?	1 Yes<br>2 No
115	Did you the respondent consented to participate in the study?	1 Yes<br>2 No
116	If no, please specify the reason	________________________________ ***END***
117	Interview start time	|___|___|:|___|___|
118	Interview end time	|___|___|:|___|___|
119	Unique identification number to be assigned by data management officer (designation code – health facility code – district code – participant code	|___|___|/ |___|___|/ |___|___| / |___|___|

Theme	Intervention component	Mechanism	Outcome	Question	Logic
	Thank you for consenting to be part of this evaluation. As mentioned, the purpose of this interview is to understand your perspective about different components of SDMC intervention that are being implemented in your health facility. I will start by asking some questions about SDMC training that you had an attended few months ago.				
1	Training on SDMC	The increased knowledge, favourable attitude, developed skills will help them provide SDMC	Applicable of knowledge and skills in care provision	1.1 Did you feel any change in the way you work at the health facility after getting SDMC training?<br>*Probe: For example, the way you deal with your patients or deal with your colleagues?*<br><br>1.2 If yes, could you tell me in detail about those changes you have experiences?<br><br>1.3 In your opinion, how the learnings that you acquired from the SDMC training influenced your work?<br>*Probe: For example, the way you deal with your patients or deal with your colleagues?*	Outcome<br><br>Outcome<br><br>Mechanism
				1.4 Would you like to propose any changes to the SDMC training?<br>*Probe: content, duration, relevance, practicality*<br><br>1.5 If yes, what changes would you like to suggest to the SDMC training?<br>Probe: content, duration, relevance, practicality<br><br>1.6 Please also tell how these proposed changes will help provision of SDMC?	Outcome<br><br>Mechanism
				1.7 What did you like about the SDMC training that was influential in your work? Please give an example.	Mechanism-Outcome

Theme	Intervention component	Mechanism	Outcome	Question	Logic
	Now I will ask some questions about assessment of participant vulnerabilities. The questions that are asked from participants at the time of admission to screen for any mental condition or disability.				
2	Assessment for the vulnerabilities	Enable maternity staff to understand the needs of pregnant women to develop care plan	Care is provided according to the identified vulnerabilities	2.1 In your opinion, has there been any changes in care provision based on vulnerability assessment?<br>2.2 If yes, could you explain in detail about those changes?	Outcome
				2.3 If any, what difficulties were faced in assessment of vulnerabilities?<br>Probe: content of questions, patient conditions, workload etc.	Mechanism
				2.4 Would you like to propose any changes to vulnerability assessment?<br>*Probe: reduce questions, add questions, merging with other registers, clear instructions*<br><br>2.5 If yes, what changes would you like to suggest to vulnerability assessment? *Probe: reduce questions, add questions, merging with other registers, clear instructions*<br><br>2.6 Please also tell how these proposed changes will help provision of SDMC?	Outcome
				2.7 Would you like to continue with the vulnerability assessment? If so, why would you like this to be continued? If no, why not?<br>Probe: personalised care, brief tools, meeting patient needs<br><br>2.8 How can we make the better use of vulnerability assessment in providing SDMC?	Outcome<br><br><br><br>Mechanism-outcome

Theme	Intervention component	Mechanism	Outcome	Question	Logic
	Now I will ask some questions about the changes in care provision with respect to maintaining patients dignity, confidentiality, information sharing, consented care etc.				
3	Benchmarking dignified care	Learning from training, motivation from posters, and results of vulnerability assessment will enable maternity team	Provision of RMC care: respect and dignity, information sharing, confidentiality, autonomy	3.1 What changes have occurred in the care provision with respect to patient dignity?<br>*Probe: engagement of companion, avoid verbal abuse, information sharing etc.*	Outcome
				3.2 In your opinion, were all maternity team adhering to the protocol of ensuring patient respect and dignity? If no, which cadre of staff members were non-adherent? And why?	Context
				3.3 Under what circumstances, is it difficult for your ensure respect and dignity of pregnant women? And what can be done to address these issues?<br>*Probe: I'm asking about scenarios when it was difficult to maintain privacy, taking consent for the procedure, avoid physical or verbal abuse, ineffective communication etc.*	Context
				3.4 What motivates you and others to provide SDMC?	Mechanism

Theme	Intervention component	Mechanism	Outcome	Question	Logic
	Now I will ask some questions about an activity that was introduced to gather women's experiences of SDMC. We would to understand the impact of this activity on your services.				
4	Assessment of women's SDMC experience	Identified strengths and deficiencies will inform improvements in care provision	Corrective actions to improve SDMC	4.1 Has there been any impact of gathering women's experiences of SDMC in quality improvements?<br>Probe: gaps in care provision, expectation and experiences of patients<br>4.2 If yes, could you explain in detail about those changes that are driven by women SDMC experiences?	Outcome
				4.3 If any, what difficulties were faced in gathering women's experiences of SDMC data? *Probe: long questionnaire, busy staff, patient not willing to respond, bias responses*	Context
				4.4 Would you like to propose any changes to assessment of women's experiences of SDMC?<br>*Probe: reduce questions, add questions, merging with other registers, clear instructions*<br><br>4.5 If yes, what changes would you like to suggest to assessment of women's experiences of SDMC? *Probe: reduce questions, add questions, merging with other registers, clear instructions*<br><br>4.6 Please also tell how these proposed changes will help provision of SDMC?	Mechanism
				4.7 Would you like to continue with assessment of women's experiences of SDMC? If so, why would you like this to be continued? If no, why not?<br>Probe: gaps in care provision, expectation and experiences of patients, brief tool<br><br>4.8 In your opinion, how can we further improve the effectiveness of women's SDMC assessment?<br>*Probe: external interviewer, increase frequency, short questionnaire*	Outcome<br><br>Mechanism-outcome

The me	Intervention component	Mechanism	Outcome	Question	Logic
	Similarly, as part of SDMC intervention we introduced another activity to document patients complaints. Now I will some questions about it.				
5	Patient complaint mechanism	Identified systemic and staff interpersonal issues will inform improvements in routine processes	Corrective actions to improve SDMC	5.1 In your opinion, has there been any changes in care provision based on patient complaint?<br>Probe: ensuring accountability, identifying gaps in services<br>5.2 If yes, could you explain in detail about those changes?	Outcome
				5.3 If any, what difficulties were faced in managing patient complaint system?<br>Probe: bias, workload, reluctance of staff	Mechanism
				5.4 Would you like to propose any changes to patient complaint system?<br>*Probe: reduce questions, add questions, merging with other registers, clear instructions, external or independent person to document*<br><br>5.5 If yes, what changes would you like to suggest to patient complaint system? *Probe: reduce questions, add questions, merging with other registers, clear instructions, external or independent person to document*<br><br>5.6 Please also tell how these proposed changes will help provision of SDMC?	Mechanism
				5.7 Would you like to continue with the patient complaint system? If so, why would you like this to be continued? If no, why not?<br>Probe: ensuring accountability, identify gaps, workload<br><br>5.8 How can we make the better use of patient complaint system in providing SDMC?	Context<br><br>Mechanism

Theme	Intervention component	Mechanism	Outcome	Question	Logic
	Now I will ask some questions about a monthly performance review meeting that was introduced in the health facility. We would to understand the impact of these monthly meetings on your work.				
6	Performance review meetings	A forum for improved communication and coordination among team members regarding work	Improved accountability and motivation for staff towards corrective actions	6.1 In your opinion, has there been any impact of monthly performance review meeting on your work?<br>Probe: ensuring accountability, identifying gaps in services<br><br>6.2 If yes, could you explain in detail about those impact?	Outcome
				6.3 If any, what difficulties were faced in conducting monthly meeting?<br>Probe: workload, reluctance of staff	Mechanism
				6.4 Would you like to propose any changes to the monthly meeting mechanism?<br>*Probe: add more people, reward system, focal person*<br><br>6.5 If yes, what changes would you like to suggest to the monthly meeting mechanism? *Probe: add more people, reward system, focal person*<br><br>6.6 Please also tell how these proposed changes will help in your work?	Mechanism
				6.7 Would you like to continue with the monthly meetings? If so, why would you like this to be continued? If no, why not?<br>Probe: ensuring accountability, identify gaps, smoothen processes	Context
				6.8 How can we further improve the effectiveness of monthly meetings?	Mechanism

Theme	Intervention component	Mechanism	Outcome	Question	Logic
	I will now ask some question about coordination between staff members to ensure provision of SDMC.				
7	Care Coordination	Coordinated efforts among staff will ensure provision of SDMC. It will also help managing work-related stress	Improved coordination will lead to better SDMC	7.1 In your opinion, has SDMC intervention (like monthly meetings, communicating patient psychosocial needs, maternity team charter) has brought any change in communication and coordination between staff members?<br>Probe: clarification of roles and responsibilities, medical errors, timely care<br><br>7.2 If yes, could you explain in detail how staff coordination has changed?	Outcome
				7.3 If any, what changes can be made to ensure sound coordination and communication between staff members?<br><br>7.4 How those changes can be implemented that will pave the way for SDMC?	

The me	Intervention component	Mechanism	Outcome	Question	Logic
	In the end, I would now like to take your suggestions or recommendations to improve SDMC intervention and how it can be better implemented in health facilities.				
8	Suggestions and recommendations	Opportunity to suggest improvement in the SDMC model	Improved SDMC strategy	8.1 What suggestions would you like to give to improve SDMC strategy?	Outcome
				8.2 How can the routine processes of SDMC strategy be further improved or made more effective?	Mechanism
				8.3 In your opinion, largely what external factors have impeded operationalisation of SDMC strategy and how can we deal with those influencers?	Context

Supplement to: Avan BI, Hameed W, Khan B, Asim M, Saleem S, Siddiqi S. Understanding the mechanisms of change for a supportive and respectful maternity care intervention in public health facilities in Sindh, Pakistan. Glob Health Sci Pract. 2023;11(6):e2300216. https://doi.org/10.9745/GHSP-D-23-00216

SDMC-Psychosocial support

Instructions

Now I would like to discuss with you about the Psychosocial support process that you have implemented in your facility. First we will discuss your experience related to each step of psychosocial support process for the patient and then in the subsequent questions we will discuss your experiences related to psychosocial support for staff and then finally we will discuss the overall psychosocial support process for the patient and staff

Instruction for facilitator regarding posters

Participant will be shared the A-4 size print of posters of psychosocial support process that was developed for patient and staff. All steps will be quickly highlighted while mentioning the difference of implementation strategies for patients and staff. *(The posters will be helpful for question 1 related to experiences about overall implementation of psychosocial support process for the patient and staff but for rest of the questions (2-8) participants will be referred to the specific section of the poster)*

Theme	Intervention component	Mechanism	Outcome	Question	Logic
**Instruction**: Now I finally like know your opinion about **overall psychosocial process for patients and also for staff** that has been implemented in your facility					
**Information card:** A systematic psychosocial process was implemented for the patients and staff with a purpose to reduce their stress and help them cope with it.					
1	Overall psychosocial support	Barriers and facilitators in implementation of psychosocial support	Improvement of psychosocial support process	1.1: what were the factors that facilitated in implementation of psychosocial support process for patient and staff?<br>Probes:<br>-System level: support from heads, support and supervision of MHFA/SDMC officer<br>-staff level: enhanced knowledge and skill, motivation to apply learned skills<br>Patient level: patient satisfaction, increased trust and compliance	Outcome
				1.2: What were the barriers in implementation of psychosocial support process for patient and staff? Probes:<br>-System level: lack of support from heads, insufficient support and supervision from MHFA/SDMC officer, lack of acknowledgement, increased workload, lack of support from colleagues<br>-staff level: lack of motivation<br>Patient level: lack of cooperation from patient/companion	Mechanism-context
				1.3: In your opinion, how psychosocial support process for patient and staff could be improved?<br>Probes:<br>-system level: monitoring and support of heads	outcome

The me	Intervention component	Mechanism	Outcome	Question	Logic
**Instruction**: Now we will talk about implementation of psychosocial support in your facility.					
**Information card:** Once vulnerability/psychosocial assessment is done. We do the psychosocial care planning whose purpose is to ensure readiness of the patient before delivery. It includes identifying companion or health worker in case of no companion for the patient, encouraging patients to share their needs and reassuring patients regarding support from you and companion.					
2	Psychosocial care planning	Ensuring readiness of the patient prior delivery	Patient satisfaction	1.0: Has the psychosocial care planning been implemented here (if yes, how and if not they why not please give reasons)<br>1.1: Did you find any change in care provision after incorporating psychosocial care planning? Probes;<br>-Staff level: *build rapport/ increase trust on health staff,*<br>-Patient level: *promote patient's autonomy (can decide the companion of her choice), patient feels that she was listened to, clarify misconception, patient feels supported and less lonely*<br>-Companion level: *support in communication process, reduce patients' distress*	Outcome
				1.2: Did you have any experience of facing difficulties in implementation of the psychosocial care planning, if yes probes:<br>-Staff level: workload, lack of support from MHFA,<br>-Patient level: emergency situation, patient was not interacting<br>-Companion level: lack of cooperation	Mechanism-context
				1.3: In your opinion, how this psychosocial care planning be improved<br>Staff level: more support from MHFA/heads, division of workload<br>Patient and companion level: should be informed about all the steps of psychosocial support and then introduce planning as one part of it	outcome

Theme	Intervention component	Mechanism	Outcome	Question	Logic
**Instruction**: Now we will talk about psycho-education which is one of the support strategies and has been implemented in your facility.					
**Information card:** The purpose of psycho-education is to provide information to patients and their companion about the child and childbirth and also about needs (physical, mental, social and emotional) and assessed condition (depression, anxiety and disability)					
3	Psycho-education regarding childbirth and psychosocial needs and support	Builds knowledge	Patients have basic knowledge about labour and childbirth and psychosocial stress and support which reduce their distress	1.0: Has the psychoeducation been implemented here (if yes, how and if not they why not please give reasons)<br><br>1.1: Did you find any change in the care provision after incorporating psycho-education? Probes;<br>-Staff level: *increase staff's knowledge about psychosocial stressors, their impact and need for support for women, enhance rapport with patient and companion, support/supervision from MHFA/SDMC officer to continue implementation*<br>-Patient level: *build their knowledge, feel more controlled, clarify misconception,* -Companion level: *build knowledge, try to support more to reduce patients' distress*	Outcome
				1.2: Did you face any difficulties in implementing psycho-education, if yes probes:<br>-Staff level: *time taking process, workload, not encouraged by the heads, psychosocial psychoeducation should be combined with psychoeducation on labour and childbirth*<br>-Patient level: *emergency situation, patient was not interacting*<br>-Companion level: *lack of cooperation*	Mechanism-context
				1.3: In your opinion, how this psycho-education for patients be improved<br>Staff level: *more support from MHFA/heads, division of workload,*<br>Patient and companion level: *should be informed about all the steps of psychosocial support and then introduce psycho-education, add importance of why it is needed*	outcome

Theme	Intervention component	Mechanism	Outcome	Question	Logic
**Instruction**: Now we will talk about reducing stress via breathing exercise which is one of the support strategies and has been implemented in your facility.					
**Information card:** Patients and their companion were guided about breathing exercise. First they were told about the purpose of this exercise that it reduces stress and makes us calm, then they were guided about the way to breathe followed by focusing on their breathing pattern and then finally told to keep practicing it during labour and childbirth, especially whenever feel stressed.					
4	Reduce stress via breathing exercise	Learn systematic skill to reduce stress	Patients experience more sense of control and less distress	1.0: Has the breathing excercise been implemented here (if yes, how and if not they why not please give reasons)<br>1.1: Did you find any change in the care provision after incorporating complete breathing strategy as a support process? Probes;<br>-Staff level: *systematic and easy to guide, being used already, support/supervision from MHFA/SDMC officer to continue implementation*<br>-Patient level: *Reduces patients distress, patients have sense of control,*<br>-Companion level: *systematic guidance such as sharing its purpose gives meaning and encouragement to the patient and companion.*	Outcome
				1.2: Did you find any difficulties in implementation of reduce stress strategy via breathing exercise, if yes probes:<br>-Staff level: *detailed process, workload, not encouraged by the heads*<br>-Patient level: *emergency situation, patient was not interacting*<br>-Companion level: *lack of cooperation*	Mechanism-context
				1.3: In your opinion, how this breathing exercise for patients be improved<br>Staff level: *more support from MHFA/heads, division of workload,*<br>Patient and companion level: *should be informed about all the steps of psychosocial support process and add importance of why it is needed*	outcome

The me	Intervention component	Mechanism	Outcome	Question	Logic
**Instruction**: Now I would like to know your opinion about companion engagement in care process which is one of the support strategies and has been implemented in your facility.					
**Information card:** Companion engagement is part of strengthening social support, where companion is guided about 10 possible roles that they could play in providing care to the patient during labour and delivery. Its purpose is to provide support from a familiar person which can reduce patient's experience of distress.					
5	Strengthen social support (companion engagement)	Engaging companion in providing support to the patient	Companion support in care process reduces patient's experience of distress during labour and childbirth	1.0: Has the companion been engaged in care process here (if yes, how and if not they why not please give reasons)<br><br>1.1: What happens when you engage companion? /Did you find any change in the care provision process after engaging companion in care process to the patient? Probes;<br>-Staff level: *share staff's burden in support provision*, *specific roles of companion make it easy to communicate*, *support/supervision from MHFA/SDMC officer to continue implementation*<br>-Patient level: *less distressed, feel supported by a familiar person, sense of security*<br>-Companion level: *companion bridge gap between patient and staff, reinforce patient to comply the instructions,*	Outcome
				1.2: Did you find any difficulties in implementing the process of engaging companion in patient's care, if yes probes:<br>-Staff level: *too many companion roles to communicate, workload, not encouraged by the heads, couldn't find time*<br>-Patient level: *more distressed because of availability of non-preferred companion*<br>-Companion level: *lack of cooperation, followed only few roles (please list)*	Mechanism-context
				1.3: In your opinion, how companion engagement for patients be improved<br>Staff level: *more support from MHFA/heads, division of workload,*<br>Patient level: *encourage patient to request preferred companion to join who could comply with staff instructions*<br>companion level: *should be informed in the beginning about all the steps of psychosocial support and then introduce the importance of why companion support*	outcome

The me	Intervention component	Mechanism	Outcome	Question	Logic
**Instruction**: Now I would like to know your opinion about promoting everyday functioning and scheduling which is one of the support strategies and has been implemented in your facility.					
**Information card:** At the time exit after delivery, patient is also guided about different way to enhance their well-being such as engaging in pleasurable activities. Especially companion of those patients who found to have depression or anxiety based on vulnerability screening are also guided to keep supporting the patient at home as well.					
6	Promote everyday functioning and scheduling	Reinforcement of self-care for well-being of patient	Continuity of post-delivery care (at the time of exit only)	1.0: Has the promoting everyday functioning and scheduling been implemented here (if yes, how and if not they why not please give reasons)<br><br>1.1: Did you find any change in the care provision process after incorporating this strategy as a support? Probes;<br>-Staff level: *build trust between patient and staff, support/supervision from MHFA/SDMC officer to continue implementation, was easy to add few points related to well-being in postnatal care guidance*<br>-Patient level: *reassures patients that health staff care for them, patient compliance*<br>-Companion level: *engage in continuity of care and support*	Outcome
				1.2: Did you face any difficulty in implementation of this strategy, if yes probes:<br>-Staff level: *time taking process, workload, not encouraged by the heads, forgot this well-being part*<br>-Patient level: *don't listen*<br>-Companion level: *lack of cooperation*	Mechanism-context
				1.3: In your opinion, how this strategy for patients be improved<br>Staff level: *more support from MHFA/heads, division of workload, making this point mandatory,*<br>Patient and companion level: *should be informed why it is needed*	outcome

Theme	Intervention component	Mechanism	Outcome	Question	Logic
**Instruction**: Now I would like to know your opinion about referral process that has been implemented in your facility for patients who are depressed or anxious.					
**Information card:** At the time exit, patients file are checked for their vulnerability assessment especially if they have screened positive for depression and/or anxiety so that they could be referred to a nearest mental health facility for further assessment and care.					
7	Referral	Suggesting patients to see specialist in case of high score of depression/anxiety	Continuity of post-delivery care (at the time of exit only)	1.0: Has the referral of patients screened positive for depression/anxiety been implemented here (if yes, how and if not they why not please give reasons)<br><br>1.1: Did you find any change in the care provision process after incorporating referring patients to mental health facility in case of having depression/anxiety? Probes;<br>-Staff level: *enhances trust between patient and providers, support/supervision from MHFA/SDMC officer to continue implementation, contribution in promoting mental health*<br>-Patient level: *patient get proper assessment and care, reflect that staff is concerned about their well-being*<br>-Companion level: *engage in continuity of care and support*	Outcome
				1.2: Did you find any difficulties in implementing the referral of patients with depression and/or anxiety, if yes probes:<br>-Staff level: *workload, not encouraged by the heads, lack of motivation,*<br>-Patient level: *felt stigmatized, wanted staff to continue this support too*<br>-Companion level: *lack of cooperation, not supporting referring of mental health specialists*	Mechanism-context
				1.3: In your opinion, how this psycho-education for patients be improved<br>Staff level: *more support from MHFA/heads, division of workload,*<br>-Patient and companion level: *should be informed about its importance that why it is needed*	outcome

Theme	Intervention component	Mechanism	Outcome	Question	Logic
**Instruction**: Now I would like to know your opinion **psychosocial process for your colleagues/staff** that has been implemented in your facility					
**Information card:** Psychosocial support process for maternity staff was also developed and implemented in your facility whose purpose was to reduce their work related stress and prevent them from burnout.					
8	psychosocial support for colleagues	Barriers and facilitators in implementation of psychosocial support for staff	Improvement of psychosocial support process for staff	1.0: Has the psychosocial support for staff been implemented here (if yes, how and [check all steps] if not they why not please give reasons)<br><br>1.1: Did you find any change in your behaviour or behaviour of your colleagues after getting training on psychosocial support for staff? Probes;<br>-Staff level: *more conscious about stress and its impact, increased knowledge, increased skill, better communication among each other, better team work, frequent use of support strategies*<br>-System level: *more support from heads/MHFA*	Secondary outcome
				1.2: Did you find any difficulties in implementation of psychosocial support process for staff? (specify, assessment, support (breathing, problem solving strategy, strengthening social support via connecting with people at work, home and other social set-up and referral)<br>Probes:<br>-System level: lack of support from heads, lack of supervision/availability of MHFA/SDMC officer, lack of acknowledgement, increased workload, lack of support from colleagues<br>-staff level: lack of motivation of colleagues	Mechanism-context
				1.3: In your opinion, how psychosocial support process for staff could be improved?<br>Probes:<br>-system level: monitoring and support of heads	Secondary outcome
